# Effect of Celastrol on LncRNAs and mRNAs Profiles of Cerebral Ischemia-Reperfusion Injury in Transient Middle Cerebral Artery Occlusion Mice Model

**DOI:** 10.3389/fnins.2022.889292

**Published:** 2022-05-23

**Authors:** Jiandong Liu, Xiangna Guo, Lu Yang, Tao Tao, Jun Cao, Zexuan Hong, Fanning Zeng, Yitian Lu, Chunshui Lin, Zaisheng Qin

**Affiliations:** ^1^Department of Anesthesiology, Nanfang Hospital, Southern Medical University, Guangzhou, China; ^2^Department of Anesthesiology, The Affiliated Dongnan Hospital of Xiamen University, School of Medicine, Xiamen University, Zhangzhou, China; ^3^Department of Anesthesiology, The Central People’s Hospital of Zhanjiang, Zhanjiang, China; ^4^Department of Anesthesiology, Affiliated Shenzhen Maternity and Child Healthcare Hospital, Southern Medical University, Shenzhen, China

**Keywords:** celastrol, ischemic stroke, lncRNA, RNA-sequencing, inflammation

## Abstract

Celastrol plays a significant role in cerebral ischemia-reperfusion injury. Although previous studies have confirmed that celastrol post-treatment has a protective effect on ischemic stroke, the therapeutic effect of celastrol on ischemic stroke and the underlying molecular mechanism remain unclear. In the present study, focal transient cerebral ischemia was induced by transient middle cerebral artery occlusion (tMCAO) in mice and celastrol was administered immediately after reperfusion. We performed lncRNA and mRNA analysis in the ischemic hemisphere of adult mice with celastrol post-treatment through RNA-Sequencing (RNA-Seq). A total of 50 differentially expressed lncRNAs (DE lncRNAs) and 696 differentially expressed mRNAs (DE mRNAs) were identified between the sham and tMCAO group, and a total of 544 DE lncRNAs and 324 DE mRNAs were identified between the tMCAO and tMCAO + celastrol group. Bioinformatic analysis was done on the identified deregulated genes through gene ontology (GO) analysis, KEGG pathway analysis and network analysis. Pathway analysis indicated that inflammation-related signaling pathways played vital roles in the treatment of ischemic stroke by celastrol. Four DE lncRNAs and 5 DE mRNAs were selected for further validation by qRT-PCR in brain tissue, primary neurons, primary astrocytes, and BV2 cells. The results of qRT-PCR suggested that most of selected differentially expressed genes showed the same fold change patterns as those in RNA-Seq results. Our study suggests celastrol treatment can effectively reduce cerebral ischemia-reperfusion injury. The bioinformatics analysis of lnRNAs and mRNAs profiles in the ischemic hemisphere of adult mice provides a new perspective in the neuroprotective effects of celastrol, particularly with regards to ischemic stroke.

## Introduction

Ischemic stroke is one of the most common cerebrovascular diseases and is a leading cause of disability and death worldwide (Feigin et al., 2014). In China, the prevalence and incidence of ischemic stroke have been increasing over the past decade (Wu et al., 2019). Traditional treatments for ischemic stroke include endovascular thrombectomy and systemic thrombolysis, but ischemia-reperfusion (I/R) injury is inevitable while restoring blood flow to the brain. Numerous drugs were developed but also failed to show benefit in the therapy of acute ischemic stroke (Chamorro et al., 2016). Hence, there is an urgent need to develop effective neuroprotective drugs for the treatment of cerebral I/R injury. Neuroinflammation and oxidative stress play pivotal roles in the pathophysiological of cerebral I/R injury, which could be an attractive therapy strategy for stroke (Maida et al., 2020).

Celastrol is a pentacyclic triterpene isolated from the traditional Chinese herb “Thunder of God Vine” (Tripterygium wilfordii Hook F.) (Salminen et al., 2010), which exhibits diverse pharmacological activities including anti-inflammatory, anti-oxidative and neuroprotective effects (Chen T. et al., 2017). Many studies have demonstrated that celastrol exerted beneficial effects in the treatment of cancer, inflammatory diseases, neurodegenerative diseases, obesity, and diabetes (Lu et al., 2021; Xu et al., 2021). In recent years, the effect of celastrol on the central nervous system has attracted close attention. Celastrol plays a neuroprotective role in a variety of neurological disorders, including neurodegenerative diseases (Paris et al., 2010; Lin et al., 2019), traumatic brain injury (Eroglu et al., 2014) and ischemic brain injury (Li Y. et al., 2012). Increasing evidence suggests that neuroprotective effect of celastrol in cerebral ischemic injury is associated with antioxidant activity and anti-inflammation property. A previous study illustrated that celastrol dramatically relieved permanent cerebral ischemia injury in rats by downregulating the expression of p-JNK, p-c-Jun and NF-κB (Li Y. et al., 2012). Another study also demonstrated that celastrol ameliorated acute ischemic stroke induced brain injury through promoting IL-33/ST2 axis-mediated microglia/macrophage M2 polarization (Jiang et al., 2018). More recently, celastrol has been reported to exhibit anti-inflammatory and antioxidant actions in rats by targeting HSP70 and NF-κB p65 and directly binding to high mobility group box 1 (HMGB1) in cerebral I/R injury (Zhang et al., 2020; Liu D. D. et al., 2021). These results suggested that celastrol may be a promising therapeutic agent for the treatment of ischemic stroke. However, little is known regarding the neuroprotective effect and the underlying mechanism of celastrol in ischemic stroke.

Long non-coding RNAs (lncRNAs) are the largest class of RNA molecules more than 200 bp in length without protein coding ability (Wang C. et al., 2017). Recently, studies have found that the mechanisms of lncRNA function involve both transcriptional and post-transcriptional regulation (Bao et al., 2018). In the post-transcriptional level, lncRNAs regulate the gene expression either by directly influencing the RNA splicing and RNA degradation, or negatively regulating the functions of miRNA as miRNA sponge (Ebert et al., 2007). Many studies demonstrated that lncRNAs participate in many crucial physiological processes and play significant roles in the occurrence and development of various diseases, including various types of tumors, cardiovascular disorders and cerebrovascular diseases (Chen et al., 2021). Previous research has uncovered that lncRNAs play critical roles in the pathogenesis of ischemic stroke (Bhattarai et al., 2017; Bao et al., 2018). Recent study indicated that lncRNA AK005401 plays an important role in the protective effect of celastrol on ischemia-induced hippocampal damage (Wang et al., 2021). However, the function and mechanism of lncRNAs in ischemic stroke need further research.

In the present study, we established the transient cerebral ischemia model in mice and evaluated the effect of celastrol on infarction volume and neurological function. Then we analyzed the different expression profiles of lncRNAs and mRNAs in the ipsilateral hemisphere after celastrol treatment by RNA-Seq. Through bioinformatics analysis of the different expression genes, we uncover the potential role of celastrol in ischemic stroke and provide a new direction on the functions and mechanisms of lncRNAs in ischemic stroke.

## Materials and Methods

### Experimental Animals

Male C57BL/6 mice (8–10 weeks old, 22–25 g) were purchased from the Experimental Animals Center of Southern Medical University. The mice were kept in a temperature-controlled animal facility under normal light/dark cycle with free access to food and water. All animals adapted to the environment for 7 days before experiments. All animal experiments were approved by the Southern Medical University Administrative Panel on Laboratory Animal Care and conducted in accordance with the guidelines of Animal Use and Care of Southern Medical University.

### Transient Middle Cerebral Artery Occlusion Model

To induce cerebral I/R injury, a transient middle cerebral artery occlusion (tMCAO) model was performed on the mice as previously described (Luo et al., 2017).Briefly, mice were anesthetized with sevoflurane (5% for induction and 2–3% for maintenance). Following a midline cervical incision, the right common carotid artery (CCA), external carotid artery (ECA), and internal carotid artery (ICA) were carefully exposed under an operating microscope. Thereafter, a silicone rubber-coated nylon monofilament (Yushun Biological Technology Co. Ltd., Pingdingshan, China) was inserted into the ECA, and advanced to occlude the middle cerebral artery for 90 min. After 90 min occlusion, the monofilament was gently pulled out for reperfusion and the incision was sutured. Mice in sham group adopted a same surgery except the middle cerebral artery occlusion.

### Drug Administration

Adult mice were randomized into three groups (sham group, tMCAO group and tMCAO + celastrol group; *n* = 18 in each group). Celastrol (Selleckchem, Houston, TX, United States) was dissolved in 1% dimethylsulfoxide (DMSO) (Sigma-Aldrich, St. Louis, MO, United States) at the concentration of 4.5 mg/kg and injected intraperitoneally at the onset of reperfusion. The mice in sham and tMCAO groups without drug treatment were injected with the same volume of DMSO. Mice were re-anesthetized and sacrificed 24 h after tMCAO. The concentration of celastrol used in the experiment was based on the concentration reported in previous study (Chen et al., 2022).

### Infarct Size Measurements

The 2,3,5-triphenyltetrazolium chloride (TTC) (Sigma-Aldrich, St. Louis, MO, United States) staining was used to determine cerebral infarction volume. After 90 min of MCAO and 24 h of reperfusion, the mice were anesthetized with 5% sevoflurane, and their brains (*n* = 5/group) were rapidly removed and coronally cut into six slices at a thickness of 2 mm using a rodent brain matrix. The brain slices were stained with 2% TTC at 37°C for 15 min and subsequently fixed in 4% paraformaldehyde at 4°C overnight. After TTC staining, the red area indicated no infarction while the white area indicated infarction. The brain slices were scanned and the infarct size was analyzed using Image J software (National Institutes of Health, Bethesda, MD, United States) by researchers who were blinded to the study group. In order to exclude the effect of cerebral edema, the following calculation formula was used: (contralateral hemisphere area–ipsilateral non-ischemic hemisphere area)/contralateral hemisphere area × 100% (Liu M. et al., 2021). Five male mice were used in each group.

### Neurological Deficit Score

The neurological deficit scores of the mice were evaluated at 24 h after tMCAO by researchers who were blinded to the experimental groups. According to the modified Bederson score, the neurological grading scores range from 0 to 5 (0, no deficit; 1, forelimb flexion; 2, as for 1, plus decreased resistance to lateral push; 3, unidirectional circling; 4, longitudinal spinning or seizure activity; and 5, no movement) (Jin et al., 2015). Seven male mice were used in each group.

### Rotarod Test

Motor performance was accessed by accelerating rotarod test after evaluating the neurological deficit scores. The mice were trained to remain on the rotarod at a starting rotation of 5 r/min which was accelerated to 40 r/min over 60 s before the model establishment (Du et al., 2020). The mice were tested under the same accelerated conditions after 24 h of reperfusion. The entire test lasted 300 s and was performed three times for each mouse at 10-min intervals. The latency of falling off of the rod were recorded and averaged.

### Primary Cortical Neurons Cultures

Primary cortical neurons were obtained from embryonic day C57BL/6 mice and cultured as previously described (Luo et al., 2017). Briefly, pregnant mice were euthanized and the cerebral cortices of the embryos were dissected and dissociated by mild trypsinization, followed by trituration in DNAase I (Sigma-Aldrich, St. Louis, MO, United States). The cells were suspended in neurobasal medium supplemented with 2% B-27 (Gibco, Grand Island, NY, United States) and 0.5 mM Glutamax (Gibco, Grand Island, NY, United States). The single cell suspension was plated in 6-well plates precoated with poly-L-lysine, and the cell culture was kept in a humidified atmosphere of 5% CO_2_ at 37°C. Half of the culture medium was replaced every 3 days and neurons were cultured for 9 days for use in subsequent experiments.

### Primary Astrocytes Cultures

Primary cultures of astrocytes were prepared from cortices of C57BL/6 newborn mice (P1–P3). Briefly, the bilateral cortices were dissected in a sterile environment and digested with 0.25% trypsin and DNAase I for 10 min at 37°C. Subsequently, cortical fragments were suspended in Dulbecco’s Modified Eagle’s medium (DMEM)/F12 (Gibco, Grand Island, NY, United States) with 10% fetal bovine serum (Gibco, Grand Island, NY, United States). Single cell suspensions were made by repeated pipetting and the cells were incubated at 37°C in a humidified 5% CO_2_ chamber for 7 days. The culture medium was replaced twice a day.

### BV2 Microglial Cell Cultures

BV2 microglial cells, which were bought from Shanghai Gaining Biological Technology Co. Ltd., were cultured in DMEM (Gibco, Grand Island, NY, United States) with 10% fetal bovine serum at 37°C in CO_2_/air (5/95%) mixture.

### Oxygen-Glucose Deprivation and Drug Treatment

To simulate ischemia-reperfusion injury *in vitro*, primary cortical neurons, primary astrocytes and BV2 microglial cells were subjected to oxygen and glucose deprivation (OGD) followed by reoxygenation. Primary astrocytes and BV2 microglial cells were incubated with glucose-free DMEM and placed within a hypoxic chamber which was continuously maintained with 95% N_2_, 5% CO_2_, 1% O_2_ at 37°C for 5 h, while primary cortical neurons for 4 h. OGD was terminated by replacing the glucose-free DMEM to their normal culture medium in the normoxic incubator with 95% air and 5% CO_2_ for 24 h. Cells incubated in normal culture medium under a normoxic incubator were used as the normoxic control. At the same time, celastrol was applied in the culture medium with the final concentration of 0.5 μM for 24 h. In contrast, the same volume of DMSO was applied in the culture medium in the control group cells. All experiments were at least duplicated three times biologically.

### RNA Extraction

The total RNA from the ischemic hemisphere or cells was isolated with the TRIzol Reagent (Invitrogen, Carlsbad, CA, United States) according to the manufacturer’s instructions. RNA purity and concentration were evaluated by using the Nanodrop ND-2000 spectrophotometer (Thermo Fisher Scientific, Waltham, MA, United States). RNA Integrity Number (RIN) was analyzed by Bioanalyzer 2100 (Agilent, Palo Alto, CA, United States). If the RIN number is >7, it can be used for high-throughput transcriptome sequencing.

### RNA-Sequencing

A total of 5 μg RNA from ischemic hemisphere was utilized for each RNA sample. Firstly, ribosomal RNA (rRNA) was depleted by Ribo-Zero Gold rRNA Removal Kit (Illumina, San Diego, CA, United States). Secondly, the left RNAs were fragmented into short fragments using divalent cations (NEBNext^®^ Magnesium RNA Fragmentation Module, NEB, Ipswich, MA, United States) under high temperature. The complementary DNA (cDNA) was synthesized and purified. Finally, the average insert size for the final cDNA library was 300 ± 50 bp. 2 × 150 bp paired-end sequencing was performed on Illumina Novaseq™6000 (LC-Bio Technology Co. Ltd., Hangzhou, China) according to the recommended protocol.

### Quality Control

Clean reads were obtained by removing reads containing adapter, reads containing ploy-N and low-quality reads from raw data by Cutadapt (Love et al., 2014). Then sequence quality was verified using FastQC (Babraham Bioinformatics, Babraham Institute, Cambridge, United Kingdom), including the Q20, Q30, and GC-content of the clean data. The downstream analysis was done on high-quality clean data.

### Differential Expression Analysis

Cuffdiff (v2.1.1) was used to calculate fragments per kilobase million (FPKMs) of both lncRNAs and mRNAs. The differentially expressed lncRNAs and mRNAs were selected with fold change > 2 or fold change < 0.5 and *P*-value < 0.05 by DESeq2. To outline the characteristics of gene expression profiles, heatmaps and volcano plots were generated by using the R package.

### Gene Ontology and Kyoto Encyclopedia of Genes and Genomes Enrichment Analysis

Gene Ontology (GO) and Kyoto Encyclopedia of Genes and Genomes (KEGG) pathway enrichment analysis of differentially expressed genes (DEGs) was implemented with DAVID.^1^ GO analysis includes the categories biological processes (BP), cellular components (CC), and molecular functions (MF). GO terms with *P* < 0.05 were defined as significantly enriched GO terms in DEGs. Pathways with *P* < 0.05 were considered as significantly enriched pathways in DEGs.

### Soft Cluster Analysis

The soft clustering Mfuzz function is based on the fuzzy c-means algorithm of the e1071 package. The R/Bioconductor package was used for soft clustering of genes, and Mfuzz-specific clusters were selected based on gene expression trends (Yang et al., 2020). The genes were determined by default parameters and the number of clusters were 12.

### Construction of ceRNA Network

According to the competitive endogenous RNA (ceRNA) mechanism, miRNA can lead to gene silencing by binding mRNA, while ceRNA can regulate mRNA expression by competitively binding miRNA. In this study, DE lncRNAs and DE mRNAs were constructed for ceRNA network. DE mRNA was put into Starbase database (Starbase V3.0) to predict upstream miRNAs, and DE lncRNA was put into miRcode to determine the targeted miRNAs. Finally, the lncRNA-miRNA-mRNA ceRNA network formed by the intersection of the two groups of predicted miRNAs. The ceRNA network was visualized by Cytoscape (Li et al., 2014).

### Quantitative Real-Time PCR

Total RNA (1 μg) was used to synthesize cDNA using a ReverTra Ace qPCR RT Master Mix with gDNA Remover (TOYOBO, Tokyo, Japan). Quantitative real-time PCR was performed on the ABI QuantStudio 6 flex (Applied Biosystems, Carlsbad, CA, United States) using SYBR Green Realtime PCR Master Mix (TOYOBO, Tokyo, Japan). GAPDH was used as a reference gene for quantification. Each experimental group was performed in triplicate to obtain the cycle time (CT) mean and the results of the analyses were calculated using the 2-ΔΔCT equation. The primer sequences were shown in the Supplementary Table 1.

### Statistical Analysis

Data are expressed as mean ± SEM. Differences were evaluated by one-way analysis of variance (ANOVA; three or more groups). *P* < 0.05 was considered statistical significance. Statistical analyses were performed using SPSS 20.0 Statistics (IBM SPSS Statistics for Version 20.0, IBM Corp, Armonk, NY United States).

## Results

### Celastrol Reduced Infarction, and Improved Neurological Scores and Motor Function After Transient Middle Cerebral Artery Occlusion

**FIGURE 1:**
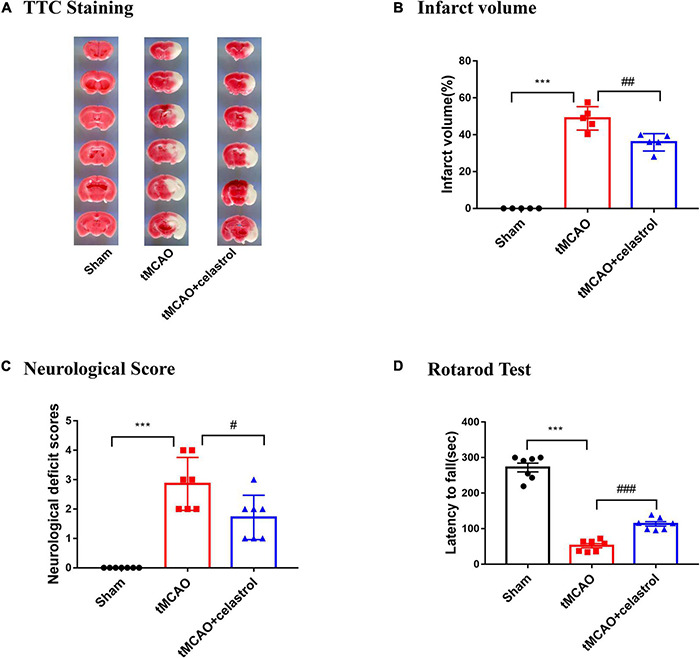
**(A,B)** Celastrol reduced infarct volume in tMCAO mice. ***P < 0.001 vs. sham group; ^##^*P* < 0.01 vs. tMCAO group, *n* = 5. **(C,D)** Celastrol improved neurological deficit scores and motor function in tMCAO mice. ***P < 0.001 vs. sham group; ^#^*P* < 0.05, ^###^*P* < 0.001 vs. tMCAO group, *n* = 7.

We examined whether celastrol improve infarct volume and neurological behavior after tMCAO. After 90 min occlusion, celastrol was immediately injected at a concentration of 4.5 mg/kg at the beginning of reperfusion. Infarct volume was measured with TTC staining after 24 h of reperfusion. The results show that the infarct volume was apparently larger in the tMCAO group (48.87 ± 2.86%) compared with sham group (0%), whereas celastrol treatment significantly reduced I/R-induced infarct volume to 35.89 ± 2.10% (Figures 1A,B). Similarly, the neurological deficit scores of mice in the tMCAO group were significantly increased to 2.85 ± 0.34, but celastrol treatment decreased neurological outcomes to 1.71 ± 0.28 (Figure 1C). Subsequently, the mice were subjected to the Rotarod fatigue test. The results showed that the time spent on the rotarod of mice in tMCAO group was 52.21 ± 5.60 s, while celastrol treatment significantly increased the time spent on the rotarod to 113.40 ± 6.40 s (Figure 1D).

### RNA-Seq Analysis of Ischemic Hemisphere After Celastrol Post-treatment in Transient Middle Cerebral Artery Occlusion

**FIGURE 2:**
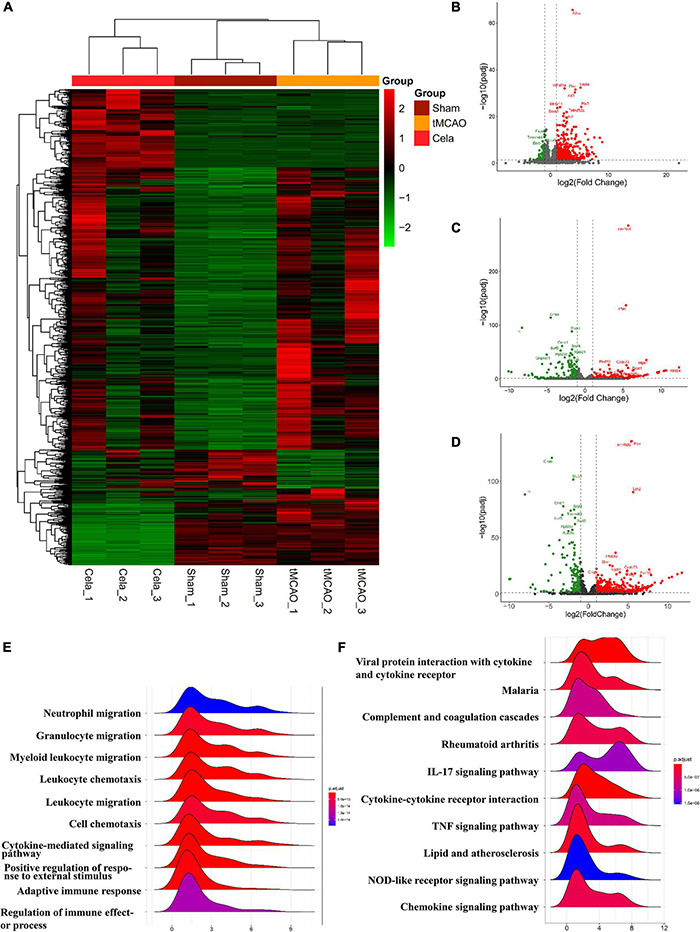
**(A)** The heatmap of DE lncRNAs and DE mRNAs. **(B–D)** The volcano plot of the mRNAs between sham and tMCAO group, tMCAO and tMCAO + celastrol group, sham and tMCAO + celastrol group. **(E)** Top 10 enrichment biological processes of GO analysis of DE mRNAs between the sham and tMCAO group. **(F)** Top 10 enrichment pathways of KEGG pathway analysis of DE mRNAs between the sham and tMCAO group.

To explore the mechanism underlying the neuroprotective function of celastrol in tMCAO, RNA-sequencing analysis was performed. Gene expression profiles for sham, tMCAO and tMCAO + celastrol groups were visualized as heatmap (Figure 2A). The fold change (FC) > 2 or FC < 0.5 and *P* < 0.05 were used as the threshold to identify the DEGs between each two groups. A total of 50 DE lncRNAs (Supplementary Table 2) and 696 DE mRNAs (Supplementary Table 3) were identified between the sham and tMCAO group. And a total of 544 DE lncRNAs (Supplementary Table 4) and 324 DE mRNAs (Supplementary Table 5) were identified between tMCAO and tMCAO + celastrol group. The mRNA profiles were further analyzed, and the distribution of mRNA was displayed by volcano plots. 612 upregulated DE mRNAs and 84 downregulated DE mRNAs were found between the sham and tMCAO group (Figure 2B). However, 168 upregulated DE mRNAs and 156 downregulated DE mRNAs were found between the tMCAO and tMCAO + celastrol group (Figure 2C). The distribution of mRNA in sham and tMCAO + celastrol group was also shown in Figure 2D.

### The Gene Ontology and Kyoto Encyclopedia of Genes and Genomes Enrichment Analysis of Differentially Expressed mRNAs

Gene Ontology and KEGG enrichment analysis were performed with DE mRNAs between sham and tMCAO groups. The top 10 results of GO analysis in the biological process on mRNA are shown in Figure 2E, including neutrophil migration, granulocyte migration, myeloid leukocyte migration, leukocyte chemotaxis, leukocyte migration, cell chemotaxis, cytokine-mediated signaling pathway, positive regulation of response to external stimulus, adaptive immune response, and regulation of immune effect or process. The top 10 results of the KEGG analysis of DE mRNAs also appeared in Figure 2F, including viral protein interaction with cytokine and cytokine receptor, malaria, complement, and coagulation cascades, rheumatoid arthritis, IL-17 signaling, cytokine-cytokine receptor interaction, TNF signaling pathway, lipid and atherosclerosis, NOD-like receptor signaling and chemokine signaling pathway. The results above indicated the potential connection between these pathways and the effects of celastrol. The results above indicated that the neuroprotective effects of celastrol are potentially related to these biological processes and metabolic pathways.

### Gene Ontology and Kyoto Encyclopedia of Genes and Genome Pathway Analysis of Two Typical mRNA Clusters

**FIGURE 3:**
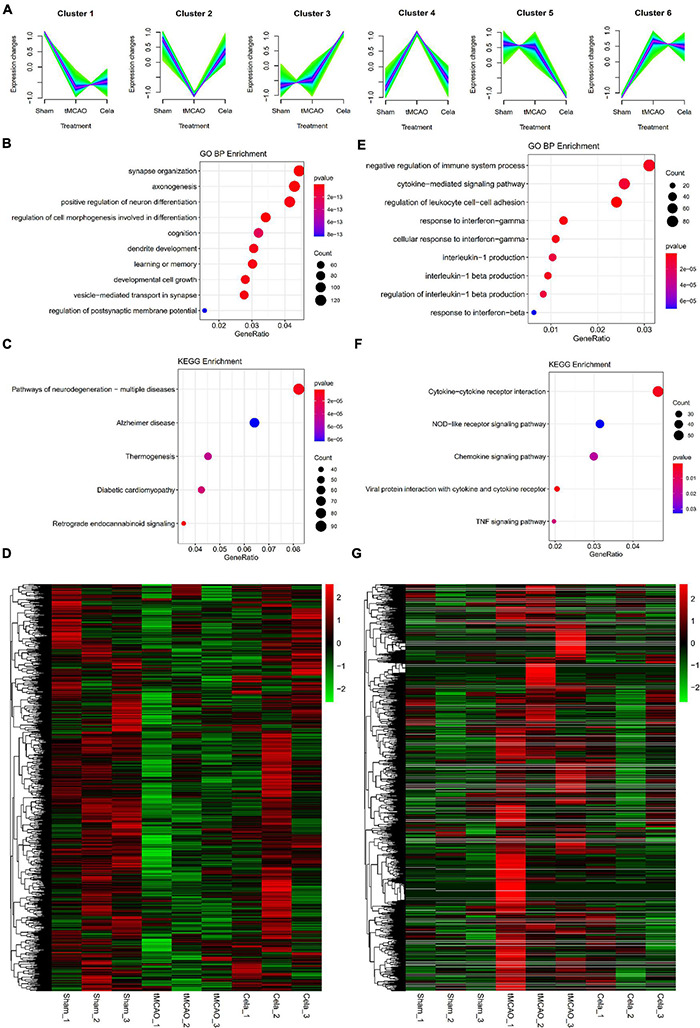
**(A)** Six clusters of mRNAs among the three groups by Mfuzz analysis. **(B–D)** The enrichment results of GO analysis, KEGG pathway and the heatmap of up-regulated genes in mRNA cluster 2. **(E–G)** The enrichment results of GO analysis, KEGG pathway and the heatmap of down-regulated genes in mRNA cluster 4.

The soft clustering method was used to assign genes to several clusters based on expression patterns. For mRNA, a total of six clusters were obtained by Mfuzz analysis in the three groups (Figure 3A). These six clusters could be classified into two large classes. One type of clusters showed upregulation of gene expression between the tMCAO and tMCAO + celastrol group (including cluster 1, 2, and 3), while another showed the downregulation. According to the experimental design, cluster 2 and cluster 4 were selected for further analysis. In order to gain insight into the biological function, GO and KEGG enrichment analysis were performed in these two clusters. The top 10 results of GO analysis in the biological process on cluster 2 and cluster 4 are shown in Figures 3B,E respectively. The results showed that genes in these two clusters were associated with different biological processes. Genes in Cluster 2 were mainly associated with neuro-related processes, such as synapse organization, axonogenesis and neuron differentiation. Genes in Cluster 4 were mainly associated with immune and inflammation. The top 5 results of the KEGG analysis also appeared in Figures 3C,F respectively. In addition, the expression of mRNA in cluster 2 and cluster 4 were shown in the heatmap respectively (Figures 3D,G). The DE mRNAs from mRNA cluster 2 and cluster 4 were respectively listed in Supplementary Tables 6, 7.

### Two Typical LncRNA Clusters via Mfuzz Analysis

**FIGURE 4:**
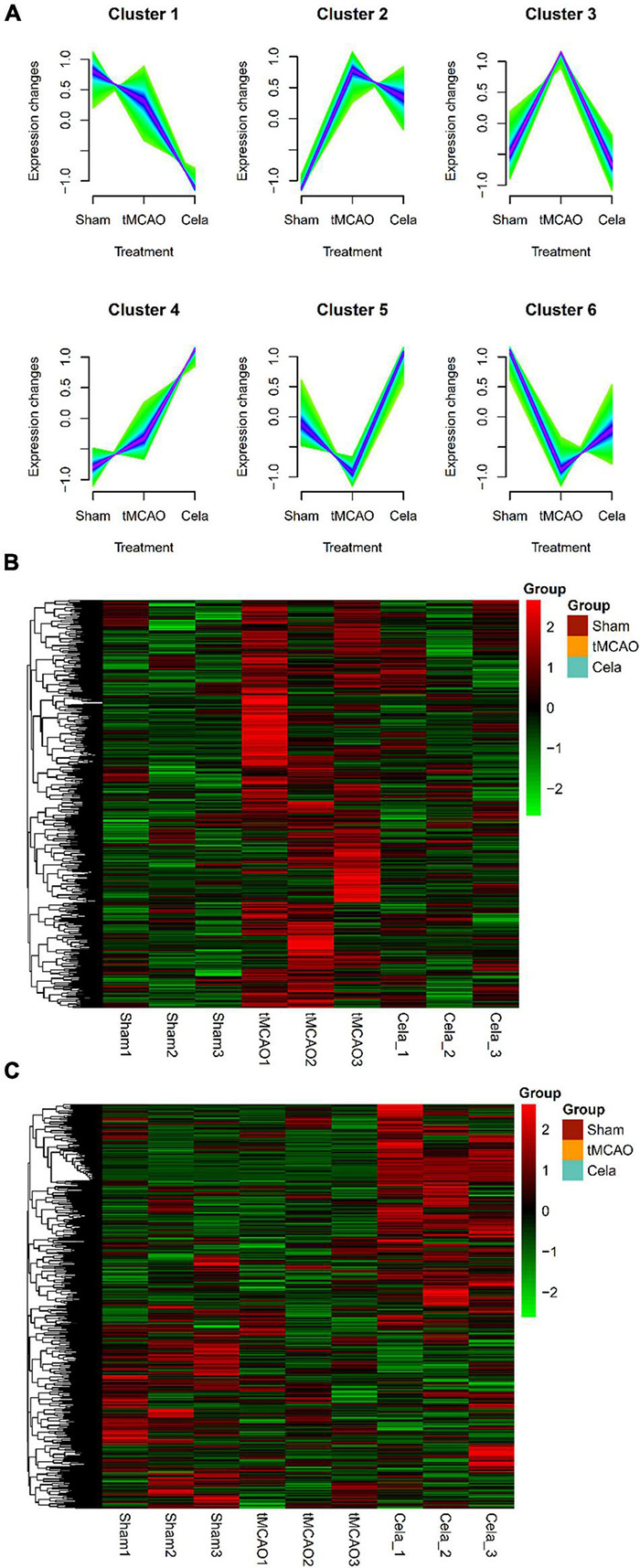
**(A)** Six clusters of lncRNAs among the three groups by Mfuzz analysis. **(B)** The heatmap of down-regulated genes in lncRNA cluster 3. **(C)** The heatmap of up-regulated genes in lncRNA cluster 5 and 6.

For lncRNA, a total of six clusters were obtained by Mfuzz analysis in the three groups (Figure 4A). These six clusters also could be classified into two large classes. One type of clusters showed downregulation of gene expression between the tMCAO and tMCAO + celastrol group (including cluster 1, 2, and 3), while another showed the upregulation. The expression of lncRNA in cluster 3 and cluster 5&6 were shown in the heatmap respectively (Figures 4B,C). The DE lncRNAs from lncRNA cluster 3 and cluster 5&6 were listed in Supplementary Table 8.

### Construction of LncRNA-MiRNA-mRNA ceRNA Network

**FIGURE 5:**
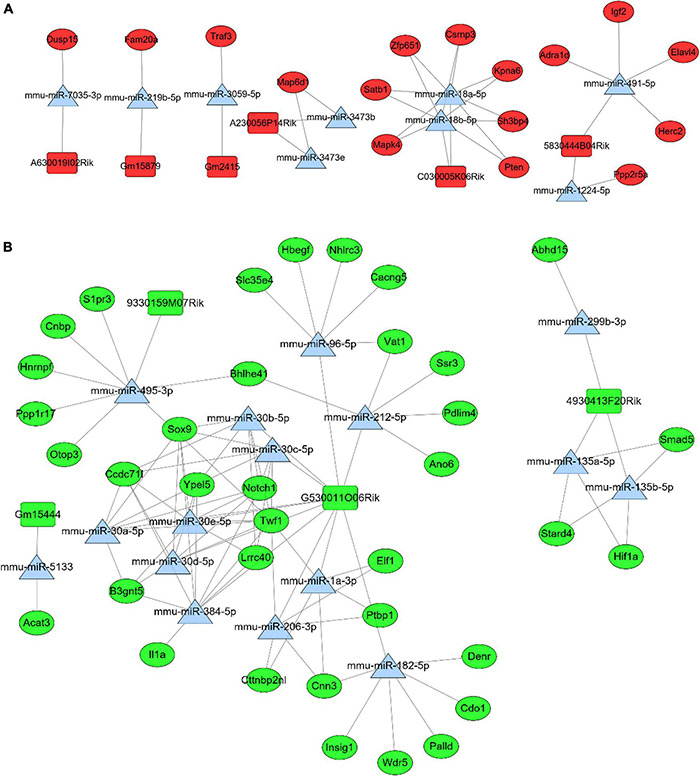
In the ceRNA network, rectangles represent lncRNAs, triangles represent miRNAs, and circles represent mRNAs. And red represents up-regulated genes and green represents down- regulated genes. **(A)** The positive regulation network of lncRNA-miRNA-mRNA ceRNA. **(B)** The negative regulation network of lncRNA-miRNA-mRNA ceRNA.

Based on differential analyses and interaction prediction, the lncRNA-miRNA-mRNA ceRNA network were established. The ceRNA networks included both positive and negative regulation (Figure 5). DE mRNAs in Mfuzz Cluster 2 (up-regulated) and DE lncRNAs in lncRNA Mfuzz Cluster 5&6 (up-regulated) were both used to predict the bound miRNAs, then the intersection of these two groups of miRNAs were formed into ceRNA network. Finally, six up-regulated lncRNAs, nine down-regulated miRNAs, and 16 up-regulated mRNAs were constructed into lncRNA-miRNA-mRNA ceRNA networks (Figure 5A). Similarly, DE mRNAs in Mfuzz Cluster 4 (down-regulated) and DE lncRNAs in lncRNA Mfuzz Cluster 3 (down-regulated) were used to predict the bound miRNAs, then the ceRNA network formed by intersection of these two groups of mirnas. Finally, ceRNA networks were constructed by 4 up-regulated lncRNAs, 16 down-regulated miRNAs and 36 up-regulated mRNAs (Figure 5B). The network consists of lncRNAs (rectangles), miRNAs (triangles), and mRNAs (circles). The red pots represent up-regulated RNAs and the green pots represent down-regulated RNAs. Above of all indicated potential critical RNA interactions involved in celastrol treatment.

### LncRNA-mRNA Interaction Network

**FIGURE 6:**
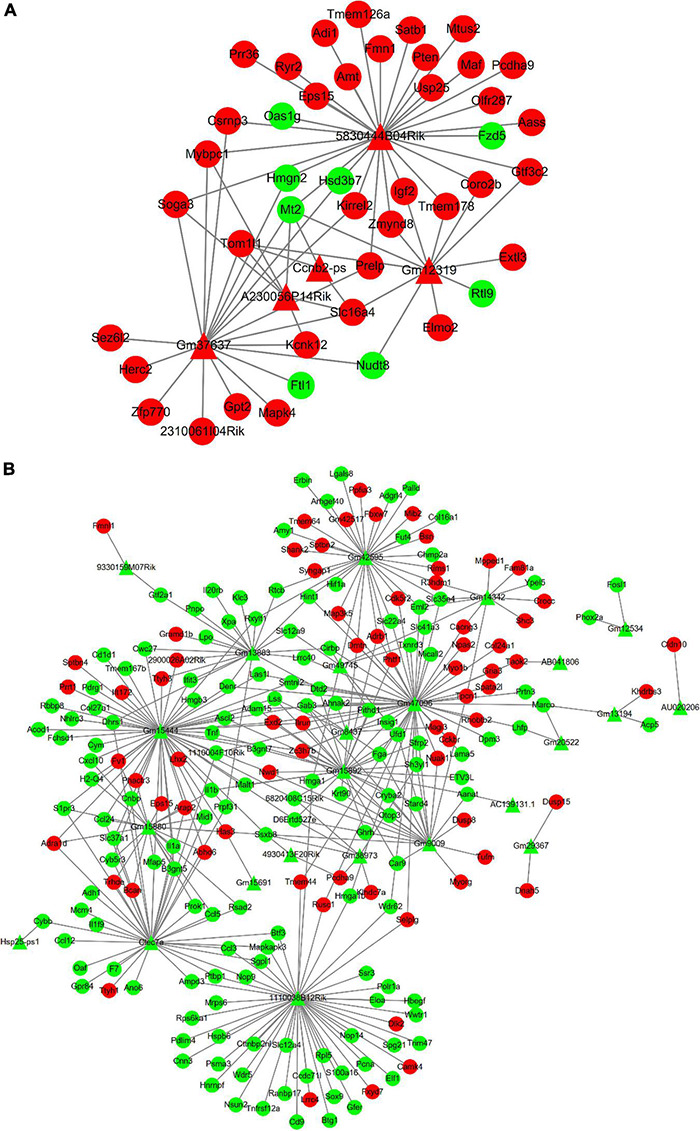
In the interaction network, triangles represent lncRNAs and circles represent mRNAs. And red represents up-regulated genes and green represents down-regulated genes. **(A)** The interaction network between up-regulated DE lncRNAs and DE mRNAs. **(B)** The interaction network between down-regulated DE lncRNAs and DE mRNAs.

The relationship between lncRNAs and mRNAs was based on the *Cis* and *Trans* function. Figure 6A shows the interaction network of differentially expressed up-regulated lncRNAs with DE mRNAs which is also the target genes of lncRNAs. Similarly, Figure 6B shows the interaction network of differentially expressed down-regulated lncRNAs with their differentially expressed target mRNAs. The Triangle node and the round node respectively represent lncRNAs and mRNAs. The red color reveals that the lncRNAs or mRNAs are significantly up-regulated, otherwise indicated with a green color.

### Validation of the Selected Differentially Expressed Genes

**FIGURE 7:**
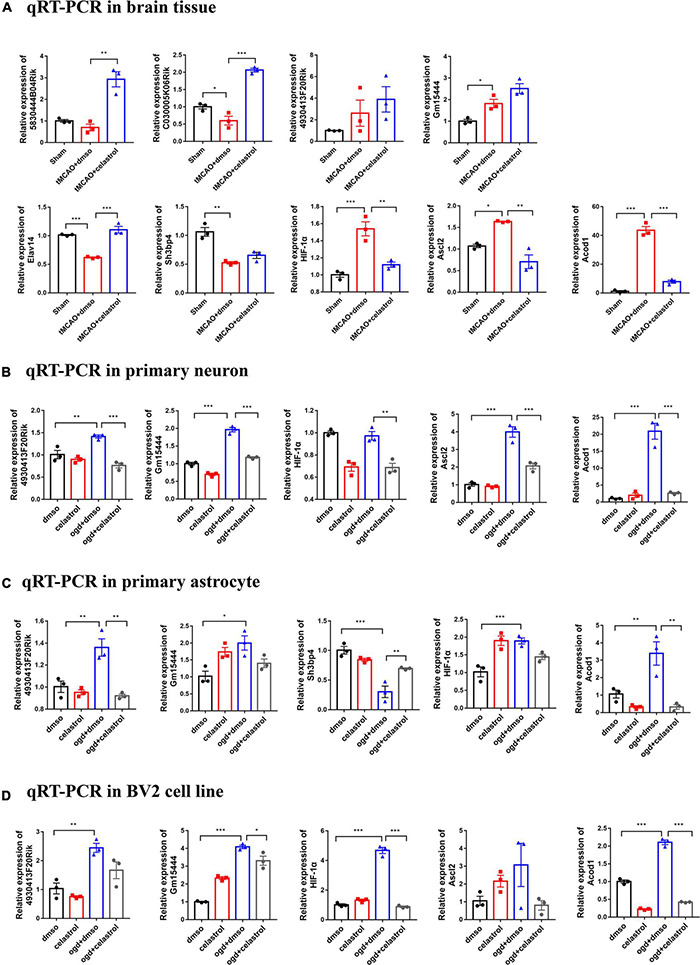
Differentially expressed lncRNAs and mRNAs confirmation by qRT-PCR. **(A)** Validation of DE lncRNAs and DE mRNAs in brain tissue. **(B)** Validation of DE lncRNAs and DE mRNAs in primary neuron. **(C)** Validation of DE lncRNAs and DE mRNAs in primary astrocyte. **(D)** Validation of DE lncRNAs and DE mRNAs in BV2 cells. *P < 0.05, **P < 0.01, ***P < 0.001, *n* = 3.

Based on the above bioinformatic analysis, we selected several DE lncRNAs and DE mRNAs for qRT-PCR validation. 5830444B04Rik-Elavl4, C030005K06Rik-Sh3bp4, and 4930413F20Rik- HIF-1α were selected from the ceRNA network, and Gm15444-Ascl2/Acod1 was selected from the DE lncRNA-DE mRNA interaction network. They were selected for further validation in the brain tissue, primary neurons, primary astrocytes, and BV2 cells by qRT-PCR. In the brain tissue, 5830444B04Rik, C030005K06Rik, Elavl4, Sh3bp4, HIF-1α, Ascl2, and Acod1 showed the same fold change patterns as those in the RNA-Seq results (Figure 7A). The expression of 4930413F20Rik and Gm15444 were consistent with lncRNA cluster 3, and Ascl2, and Acod1 were also consistent with mRNA cluster 4 in three types of cells. The expression of HIF-1α was consistent with mRNA cluster 4 both in the primary astrocyte and BV2 cells and the expression of Sh3bp4 was consistent with mRNA cluster 2 in the primary astrocyte (Figures 7B–D). But some of them did not reach statistical significance. The validation of the other DEGs were shown in the Supplementary Figure 1.

## Discussion

In present study, we found that celastrol reduced the cerebral infarction volume in mice, improved the neurological and motor function. These results are consistent with the previous studies, which demonstrated that celastrol post-treatment reduces ischemic stroke-induced brain damage in rats (Li Y. et al., 2012; Jiang et al., 2018; Liu D. D. et al., 2021). Biological process of GO and KEGG pathway analysis of DE mRNAs suggested that inflammation and immunity response play important roles in the ischemia stroke. Neuroinflammation is involved in the pathophysiological process of ischemia reperfusion injury and anti-inflammatory is an important target for the development of neuroprotective drugs (Maida et al., 2020). Four lncRNAs and five mRNAs were selected from the lncRNA-miRNA-mRNA ceRNA network and lncRNA-mRNA co-expression network for further validation by qRT-PCR and most of them showed the same fold change patterns in brain tissue as those in the RNA-Seq results. Taken together, these results suggest that DEGs may play a complicated role in the neuroprotective effect of celastrol in ischemic stroke, and the potential function of them required further validation and annotation.

The potential mechanism of celastrol in neuroprotective effect deserves further investigation. Celastrol is the most abundant compound extracted from the root of Tripterygium wilfordii (Salminen et al., 2010), which has exhibited preclinical and clinical efficacy in a broad range of diseases such as cancer, rheumatoid arthritis (Tao et al., 1989), ulcerative colitis (Gao et al., 2020), and central nervous system disease (Schiavone et al., 2021) due to its different pharmacological properties. Celastrol was demonstrated to exert anticancer effects in many types of tumors such as breast cancer (Yang et al., 2011), retinoblastoma (Li Z. et al., 2012), gastric cancer (Sha et al., 2015), and melanoma (Abbas et al., 2007) by suppressing tumor migration, invasion and angiogenesis as well as promoting autophagy and apoptosis. Hsp90, NF-κB, HIF-1α/VEGF, PTEN/PI3K/Akt, Akt/mTOR, and ROS/JNK signaling pathways were identified as relevant anticancer targets and underlying mechanisms of celastrol (Lu et al., 2021). In the past several decades, the anti-inflammatory effects and mechanisms of celastrol also became clearer. Several studies showed that celastrol can alleviate rheumatoid arthritis by inhibiting inflammatory cytokines and oxidative stress as well as regulating the calcium homeostasis (Cascão et al., 2012; Li et al., 2013; Wong et al., 2019). Furthermore, Shaker et al. demonstrated celastrol can ameliorate inflammatory symptoms in mice colitis model, and the relevant mechanism involved the inhibition of the NOD-like receptor protein 3 inflammasome (NLRP3-inflammasome), reduction of the levels of IL- 23 and IL-17A as well as the up-regulated expression of IL-10 and TNF-α (Shaker et al., 2014). As a potent inhibitor of inflammation and oxidative stress, celastrol was confirmed to have a potential neuroprotective effect in central nervous system disease, such as neurodegenerative diseases (Konieczny et al., 2014), neuropsychiatric disorders (Zhu et al., 2021) and ischemic stroke (Li Y. et al., 2012). Inflammatory insult and oxidative stress have been implicated in the pathogenesis of ischemic stroke. Recently, increasing studies demonstrated neuroprotective effects of celastrol in permanent and transient ischemic stroke in rodents (Jiang et al., 2018; Zhang et al., 2020). However, owing to the complexity of the underlying signaling pathways, further effort is needed to further illustrate the neuroprotection mechanism of celastrol in ischemic stroke. In this study, we found “TNF signaling pathway” and “NOD-like receptor signaling pathway,” the two signaling pathways are both significantly enriched in the ischemic stroke and celastrol post-treatment process. And the immune response such as “negative regulation of immune system process” and “response to interferon-gamma and interferon beta” also contribute to the transcription profile change of celastrol. These results suggests that anti-inflammatory effect and immune response of celastrol may be the main factor of reducing cerebral ischemia-reperfusion injury, which is consistent with previous studies. What’s interesting, the KEGG pathway analysis also suggested that lipid metabolism may play a potential role in neuroprotection of celastrol. More recently, a study found lipid metabolism partially regulated the neuroprotection of celastrol on cerebral I/R injury through the lipidomic analysis (Liu M. et al., 2021), but the underlying mechanism need further investigation.

It is essential to explore the potential targets of celastrol in ischemia stroke. Although previous study has found that the neuroprotective action of celastrol was partly due to its inhibition of neuroinflammation through directly binding with HMGB1 protein (Liu D. D. et al., 2021), it remains essential to explore more direct targets of celastrol. In this study, we selected five DE mRNAs from the ceRNA network or lncRNA-mRNA interaction network for the further validation by qRT-PCR, including Elavl4, Sh3bp4, HIF-1α, Ascl2, and Acod1. In the brain tissue, almost all of them showed the same fold change patterns as those in the RNA-Seq results. Among them, HIF-1α, Acod1, and Elavl4 attracted our great attention. First, hypoxia inducible factor-1 alpha (HIF-1α) is the main subunit of hypoxia-inducible factor, which is an oxygen-dependent transcriptional activator (Lee et al., 2004). Accumulating evidence elucidated that HIF-1α plays an important role in suppressing oxidative stress and inflammation in stroke (Baranova et al., 2007; Amin et al., 2021). In this study, we found that the expression of HIF-1α was consistent with mRNA cluster 4 in mice brain tissue, primary astrocyte and BV2 cells. Combined with previous studies, how celastrol alleviates cerebral ischemia-reperfusion injury via HIF-1α need further study. Secondly, aconitate decarboxylase 1 (ACOD1, also known as immune-responsive gene 1 [IRG1]), has attracted much attention as a multifunctional regulator of immunometabolism in inflammation and infection (Michelucci et al., 2013; Cordes et al., 2016). ACOD1 plays important roles in many diseases by regulating itaconate production, oxidative stress, and inflammation (Wu et al., 2020). ACOD1-mediated itaconate has been demonstrated to play an anti-inflammatory role in macrophages. Recently, ACOD1 has been proved to have an association with neurotoxic microglial activation and chronic neuroinflammation (Lampropoulou et al., 2016). More recently, a study demonstrated that Acod1 KO mice presented significant increase in cerebral lesion volume compared to control mice and illustrated that ACOD1 suppressed cerebral ischemia-reperfusion injury by oxidative stress regulation (Lampropoulou et al., 2016). In present study, we found the expression of ACOD1 presented up-regulation both in the tMCAO mice and three types of cells with OGD/R, which was reversed by celastrol. The role of ACOD1 in cerebral ischemic stroke and how does celastrol regulate ACOD1 have attracted much attention. Thirdly, ELAVL4 (also known as HuD), an RNA-binding protein, is mainly expressed in neuronal systems and regulates the metabolism of target mRNAs (McKee and Silver, 2007; Singh et al., 2015). ELAVL4 plays important roles in neuronal processes, including neuronal development, differentiation, dendritic maturation, and neural plasticity (Akamatsu et al., 2005; Bronicki and Jasmin, 2013). Several studies have shown that ELAVL4 involved in the pathogenesis of neurodegenerative diseases, such as Alzheimer’s disease (Kang et al., 2014), Parkinson’s disease (Noureddine et al., 2005), and amyotrophic lateral sclerosis (Dell’Orco et al., 2021). However, whether ELAVL4 is involved in the pathogenesis of ischemic stroke remains unclear. In the brain tissue, ELAVL4 showed the same fold change patterns as those in the RNA-Seq results. These results suggested that celastrol may participate in the regulation of ischemic stroke, but the underling mechanism and whether celastrol can play a neuroprotective role through ELAVL4 need further study.

Further studies on lncRNAs are beneficial to reveal the underlying mechanism of celastrol in cerebral I/R injury. LncRNAs play important roles in brain development, neuron function, neuronal proliferation and apoptosis (Briggs et al., 2015). Numerous studies have demonstrated that lncRNAs are engaged in the occurrence and development of various central nervous system diseases, such as Alzheimer’s disease (AD), Parkinson’s disease (PD), Huntington’s disease (HD), and ischemic stroke (Bao et al., 2018; Vangoor et al., 2021). Increasingly evidence has elucidated that lncRNAs play critical role in the pathogenesis of ischemic stroke. A bulk of aberrantly expressed lncRNAs have been reported in ischemic stroke patients (Dykstra-Aiello et al., 2016), rodent stroke models (Wang J. et al., 2017) or oxygen-glucose deprived (OGD) cells (Zhang et al., 2016) by RNA-seq and microarrays. Notably, Yin at al. confirmed that Malat1 was involved in the protection of cerebral microvasculature and parenchyma after cerebral ischemic insults through inhibiting endothelial cell apoptosis and inflammation. Moreover, Malat1 KO mice appeared larger cerebral infarct size, worsened neurological deficit, and weaken sensorimotor functions (Zhang et al., 2016, 2017). Other lncRNAs, such as ANRIL (Bai et al., 2014), SNHG14 (Qi et al., 2017), TUG1 (Chen S. et al., 2017), and MEG3 (Yan et al., 2016), were also found to affect neuronal apoptosis, inflammation and angiogenesis during ischemic stroke. More recently, Zhang at al. indicated celastrol can reduce I/R-mediated hippocampal injury by downregulating AK005401/MAP3K12 signaling, and its neuroprotection was alleviated by AK005401 overexpression (Wang et al., 2021). However, the above lncRNAs was not found to be the DE lncRNAs in this study, we suggested that the different species and different cerebral ischemic models can explain this problem. In current study, 5830444B04Rik, C030005K06Rik, and 4930413F20Rik from ceRNA network and Gm15444 from lncRNA-mRNA co-expression network were selected for further validation by qRT-PCR in brain tissue and three types of cells. Most of them showed the same fold change patterns as those in the RNA-Seq results, while others showed different fold change patterns probably because of the methodological or statistical differences. In order to elucidate the functions and mechanisms of celastrol on I/R-mediated neuronal injury, further study on these lncRNA is needed.

Undoubtedly, this study has several limitations. First, the RNA-seq tested the expression profile of mRNAs and lncRNAs, but without miRNAs. Second, differences in gene expression detected by RNA-seq and qPCR may be due to methodological or statistical differences. Third, the validated mRNAs and lncRNAs by qRT-PCR and bioinformatic analysis still need deliberately designed experiment to further undermine the regulating mechanism.

In conclusion, the present study demonstrated that celastrol treatment can effectively reduce cerebral ischemia-reperfusion injury. Celastrol can influence the expression of lncRNAs and mRNAs in ischemia stroke, and bioinformatics analysis have identified that inflammation related biological processes and KEGG pathways associated with celastrol treatment. Several lncRNAs or mRNAs of potential therapeutic targets were selected for further validation. Our results provide a framework for further investigation of the role of lncRNAs and their target mRNAs in the neuroprotective effects of celastrol, especially in ischemic stroke.

## Data Availability Statement

The data of RNA-sequencing in this study can be obtained from the NCBI GEO database at this link: https://www.ncbi.nlm.nih.gov/geo/query/acc.cgi?acc=GSE202659.

## Ethics Statement

The animal study was reviewed and approved by Southern Medical University Administrative Panel on Laboratory Animal Care.

## Author Contributions

ZQ, CL, and TT designed the experiments. JL, XG, LY, and JC performed the experiments. XG, ZH, FZ, and YL wrote the manuscript and analyzed the data. All authors contributed to the article and approved the submitted version.

## Conflict of Interest

The authors declare that the research was conducted in the absence of any commercial or financial relationships that could be construed as a potential conflict of interest.

## Publisher’s Note

All claims expressed in this article are solely those of the authors and do not necessarily represent those of their affiliated organizations, or those of the publisher, the editors and the reviewers. Any product that may be evaluated in this article, or claim that may be made by its manufacturer, is not guaranteed or endorsed by the publisher.

## Footnotes

1
http://david.ncifcrf.gov/
