# Supplementary figures and images for: Effect of Celastrol on LncRNAs and mRNAs Profiles of Cerebral Ischemia-Reperfusion Injury in Transient Middle Cerebral Artery Occlusion Mice Model
Source: Front Neurosci. 2022 May 23;16:889292. doi: 10.3389/fnins.2022.889292 (PMC9169531; doi:10.3389/fnins.2022.889292)

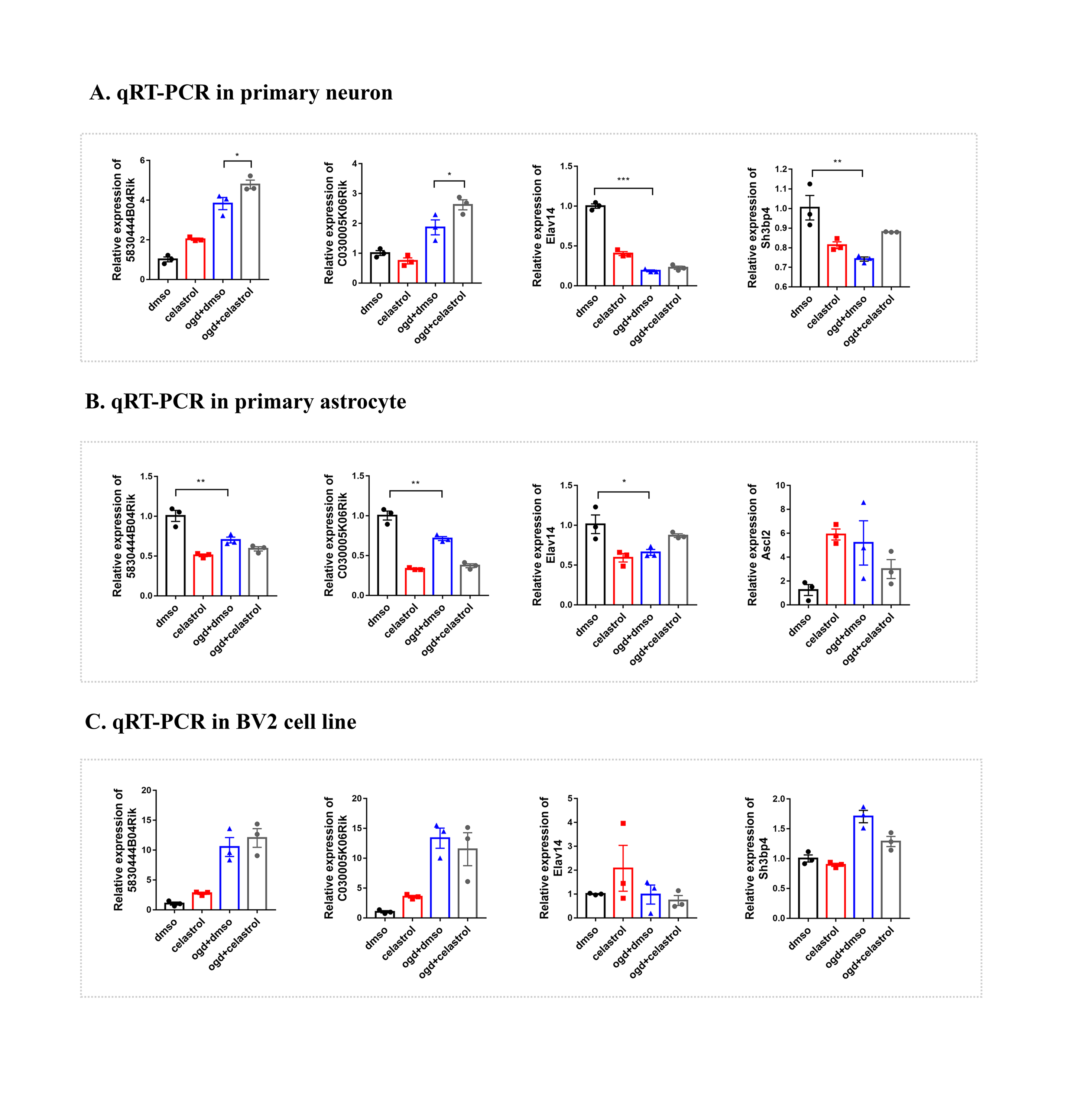

Supplement: Supplementary file 9 [file Image_1.TIF]
